# Evaluation and improvement of workplace vertical violence of nursing interns based on the Importance-Performance Analysis method

**DOI:** 10.3389/fmed.2023.1210872

**Published:** 2023-09-28

**Authors:** Weifang Xu, Lili Feng, Haohao Huang, Siqi Liu, Mao Ye, Fuqin Tang, Yen-Ching Chuang, Fuman Cai

**Affiliations:** ^1^College of Nursing, Wenzhou Medical University, Wenzhou, Zhejiang, China; ^2^Department of Orthopedics, Taizhou Central Hospital (Taizhou University Hospital), Taizhou, Zhejiang, China; ^3^Department of Nursing, Taizhou Central Hospital (Taizhou University Hospital), Taizhou, Zhejiang, China; ^4^Department of Burn Wound Repair, The First Affiliated Hospital of Wenzhou Medical University, Wenzhou, China; ^5^Department of Intensive Care Unit, Taizhou Central Hospital (Taizhou University Hospital), Taizhou, Zhejiang, China; ^6^Institute of Public Health and Emergency Management, Taizhou University, Taizhou, Zhejiang, China; ^7^Business College, Taizhou University, Taizhou, Zhejiang, China; ^8^Key Laboratory of Evidence-Based Radiology of Taizhou, Linhai, Zhejiang, China

**Keywords:** Importance-Performance Analysis (IPA), multiple criteria decision-making (MCDM), nursing interns, priority improvement, workplace vertical violence

## Abstract

**Purpose:**

To analyze the key factors related to workplace vertical violence among nursing interns in China and to propose strategies to improve the nursing practice environment.

**Methods:**

A cross-sectional study was conducted using the Importance-Performance Analysis (IPA) method to analyze the key factors and significance of workplace vertical violence for nursing interns. The data were obtained by administering a workplace vertical violence survey, designed specifically for this study, to 120 nursing interns at a tertiary general hospital in Zhejiang Province, China.

**Results:**

The results demonstrated that the variables “I was ordered to do something beyond my ability and lacked guidance (*C*_3_),” “Errors in work have been repeatedly emphasized, spread, or exaggerated (*C*_8_),” “I was unjustly criticized (*C*_9_),” “I was withheld or blocked information purposefully (*C*_1_),” and “I was belittled at work (*C*_2_)” were the most crucial variables for determining the presence of workplace vertical violence of nursing interns. Moreover, they are priority improvement variables.

**Conclusion:**

Managers must prioritize the use of relevant resources during internships to minimize false reinforcement and unfair criticism. Efforts should focus on improving information sharing, emphasizing the role of nursing interns in clinical work, providing better guidance when arranging for nursing interns to do work that exceeds their capacity, reducing workplace vertical violence, and improving nursing intern practice environments.

## Introduction

Vertical violence is a type of workplace violence that occurs between colleagues in different hierarchical positions, i.e., superiors and subordinates (1). It is particularly prevalent in the healthcare sector, where gender and professional hierarchies can often exacerbate its impact. Both male and female healthcare professionals may experience vertical violence, but the experiences and consequences can vary significantly based on gender (2). The term “workplace vertical violence” was coined from the concept of horizontal violence (3), which refers to intentional, unnecessary, or unjustifiable acts directed by one employee toward another of the same status with the intention to harm, isolate, belittle, manipulate, or undermine them (4).

Working conditions in healthcare can often be stressful and demanding, which may contribute to the occurrence of vertical violence. Extended working hours, high workload, lack of resources, and poor management are among the adverse working conditions that could lead to increased stress and frustration, thereby leading to vertical violence (5). The consequences of vertical violence are far-reaching and can impact both professionals and patients. For professionals, this type of violence can lead to decreased job satisfaction, increased stress, and burnout. For patients, it can affect the quality of care they receive, as healthcare professionals affected by violence may not be able to perform their duties effectively (6). The most common types of vertical violence in the healthcare sector include belittling, unfair criticism, excessive demands, and even physical abuse (7). However, the frequency and severity of these types can vary widely, depending on various factors such as the workplace culture and the individual personalities involved. Horizontal nurse-to-nurse violence is common in clinical settings (8–11). The phenomenon of “nurses eating their young” (12), is often used to describe workplace vertical violence. Student nurses and interns are considered to be the most vulnerable and at the highest risk of being targets (13).

In addition, nursing interns lack clinical and life experience, making them less proficient in acquiring coping skills and placing them at the bottom of the environmental hierarchy (14). Consequently, they are located at the periphery of the dominant hospital group. These factors may increase the vulnerability to vertical violence in the workplace. Most studies suggest that patients are the most common perpetrators of lateral violence, followed by friends and relatives of patients (15, 16). However, in the workplace of nurses, vertical violence typically originates with colleagues or nursing instructors (17).

Comparisons between studies can be challenging because of variations in definitions and types of clinical violence studied. For example, in a UK study, 42.18% of student participants reported experiencing bullying or harassment while on clinical placement in the previous year. Of these incidents, 30.4% involved witnessing the bullying or harassment of other students and 19.6% involved qualified nurses as the perpetrators (17). Smith et al. (18) reported that nursing interns had varying experiences of bullying in clinical practice, with the majority of the perpetrators being nursing staff and clinical instructors. Additionally, they identified seven categories of consequences associated with bullying: psychological distress, anger, fear, loss of confidence and self-esteem, and a diminished ability to learn about and provide care to patients.

Studies have shown that over half of nursing interns (51%) reported that workplace violence influenced their future career choices (19). Furthermore, among staff nurses, those who experienced workplace violence at a younger age were more likely to resign from their jobs than their older counterparts. Clinical violence also creates uncertainty about career choices among nursing interns, and those affected may consider leaving the profession altogether (8). As a cyclic phenomenon, individuals who consider horizontal violence a normative experience may potentially perpetuate and reinforce these harmful behaviors themselves in the future (20). To provide support and facilitate the transition from education to clinical practice for nursing interns and the next generation of nurses, several institutions, such as the United States, United Kingdom, Sweden and Australia Institute of Medical Research, recommend implementing transition programs (TPs) to enhance their resilience and decrease turnover rates (21, 22). Although transition programs have been effective in supporting newly graduated nurses and easing their transition, there is limited evidence of its effectiveness in reducing workplace violence, bullying, and stress. Furthermore, there is a dearth of research on nursing interns experiences in such programs.

Previous studies have mainly focused on the incidence, types, resources, and prevalence of workplace vertical infections. While four levels of injustice encountered by nursing interns have been identified, including “being unwanted and ignored,” “distrusted and disbelieved assessments,” “unfair blame,” and “public humiliation” (23), there are few studies on preventive measures. It is important to determine whether the factors contributing to workplace vertical violence differ between nursing interns and their international colleagues in different educational settings and cultures. Additionally, it is necessary to identify the types of violence that have the greatest impact on the physical and mental health of nursing interns and develop effective intervention methods.

In this study, we introduce a theoretical model that explores the impact of vertical violence on the workplace experiences of nursing interns in China. The model suggests that vertical violence directly affects interns’ job satisfaction, stress levels, and professional commitment, influencing their willingness to stay in the nursing profession. To address this issue, we utilize the Importance-Performance Analysis (IPA), a multicriteria decision analysis method. The IPA allows us to identify key factors of vertical violence and propose improvement measures, distinguishing our study from previous ones. This model and the application of IPA can guide the nursing teaching sector in developing measures to improve the working environment for nursing interns.

## Materials and methods

### Study design

This study aims to establish a Multi-Criteria Decision Making (MCDM) model and implement it within the context of nursing intern workplaces. This instrument for questionnaire on nursing students’ workplace vertical violence was adopted as a key evaluation tool, encompassing multiple subscales. Factor weights were subsequently defined using an Importance-Performance Analysis (IPA) method, providing a comprehensive view of the relative significance of each aspect. This constructed model was ultimately deployed to assess and propose enhancements to mitigate vertical violence in nursing intern environments, with an aim to bolster the overall practice setting. Since this study was aimed at the entire population, sample selection was not involved.

In this research, a cluster sampling survey was implemented to gather data from 130 nursing students who were on the verge of completing their clinical practice (duration of practice ≥8 months) at a tertiary general hospital in Zhejiang Province. Out of the 130 distributed questionnaires, 120 were entirely filled out, representing a response rate of 92.3%. The remaining 10 students who did not participate in data collection were consequently omitted from the study.

### Questionnaire on nursing students’ workplace vertical violence

The survey tool used in this study was developed by Stevenson et al. (24) for use in college nursing students in the United Kingdom. It comprises 25 statements associated with the phenomenon of bullying, on which students are asked to indicate behavior frequency based on a Likert-type scale ranging from never having experienced the bullying behavior to having experienced the bullying behavior all the time. For the purposes of Clarke et al.’s study (23), minimal modify cations were made to improve clarity, reduce redundancy, improve conciseness, and reduce potential ambiguity of answers. Each subscale showed high internal reliability, with Cronbach’s alpha coefficients ranging from 0.86 to 0.93. In 2019, Tian et al. (7), using cluster sampling method, conducted a longitudinal questionnaire survey on workplace violence among 486 nursing students, and adjusted the scale again to fit the Chinese population. In their study, the reliability coefficient of the tool’s Cronbach’s alpha was *r* = 0.971 (25). On the basis of the above literature, the researchers compiled a questionnaire on vertical violence in the workplace of nursing interns, as shown in Table 1.

**Table 1:** Questions of nursing interns’ workplace vertical violence.

Item	Content
*C*_1_	I was withheld or blocked information purposefully.
*C*_2_	I was belittled at work.
*C*_3_	I was ordered to do something beyond my ability and lacked of guidance.
*C*_4_	Others spread gossip or rumors about me.
*C*_5_	I was frozen out, ignored, or excluded.
*C*_6_	I was threatened or intimidated.
*C*_7_	I was humiliated publicly.
*C*_8_	Errors in work have been repeatedly emphasized, spread, or exaggerated.
*C*_9_	I was unjustly criticized.
*C*_10_	I was physically abused (such as pushing body behavior).
*C*_11_	I was deprived of proper rights.
*C*_12_	I was a laborer who was forced to do trivial and unimportant work.
*C*_13_	I was treated with hostility.
*C*_14_	I became a scapegoat.
*C*_15_	Turn to others for help, but they refused to help.

The first section of the questionnaire included sociodemographic information such as age, sex, school, and internship length. The second section inquired about their importance. This section was scored on a 5-point Likert scale, ranging from very unimportant (1) to very important (5). The higher the total score, the more important the violence type was in the nursing interns’ perceptions. The third section of the questionnaire involved the degree of performance, which used a reverse-scoring method. Responses ranged from completely agree (1) to completely disagree (5). The higher the score, the less likely the nursing interns encountered vertical violence at work.

### Procedure and ethical issues

Information about the participants was collected anonymously. All procedures were performed in accordance with the guidelines of the Ethics Committee of the Zhejiang Taizhou Central Hospital (Approval Number: 2023 L-02-01) and the tenets of the Declaration of Helsinki. All participants were verbally assured that their responses would be confidential. During the data collection, 120 nursing interns were present in the classroom and agreed to participate in the study.

### IPA method

This method was proposed by Martilla and James (26). The Importance-Performance Analysis (IPA) method is used to evaluate the relative importance of various factors in a system or process and to identify areas for improvement. Since then, it has been widely used in the fields of nurses job satisfaction (27, 28), shared decision-making (29), and university-teacher development (30).

**Figure 1:**
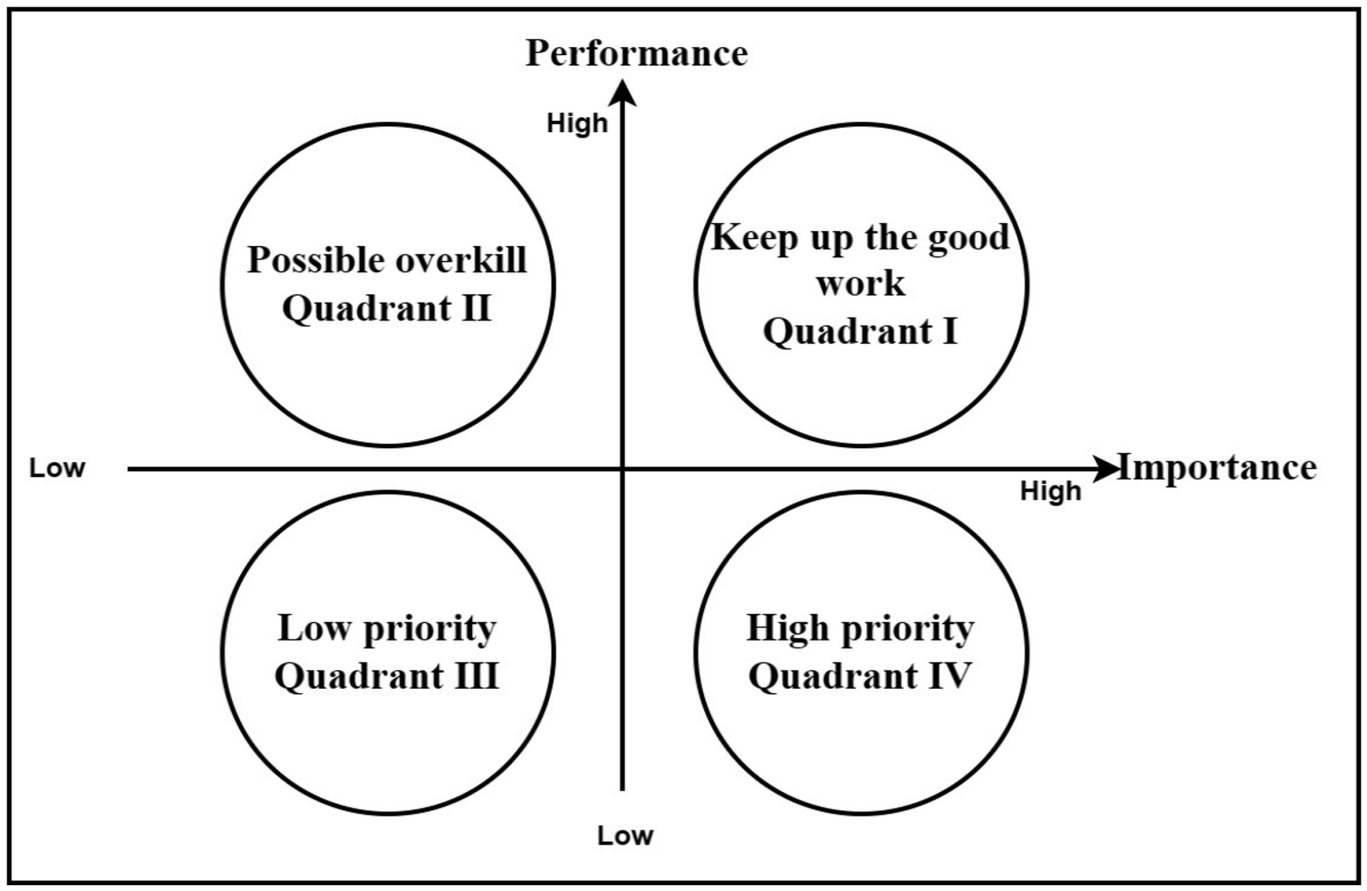
Four quadrant diagram by Important-performance analysis.

To perform an Importance-Performance Analysis (IPA), the factors being evaluated are assessed based on their importance and performance ratings, and are subsequently partitioned into four quadrants. To achieve this, a two-dimensional grid was used, with the horizontal axis representing importance and the vertical axis representing performance. The factors were then plotted on a grid according to their respective importance and performance ratings, and four quadrants were identified based on these ratings. The grid is divided into four quadrants based on a diagonal line that represents the “performance is equal to importance” thresholds, shown in Figure 1. The four quadrants were defined as follows:

Maintaining good work (Quadrant I): This quadrant includes factors that are highly important and have a high level of performance. These factors are already performing well and should be maintained at their current levels.

Possible overkill (Quadrant II): This quadrant includes factors that are of low importance, but have a high level of performance. These factors may consume resources that are better allocated to the other areas.

Low priority (Quadrant III): This quadrant includes factors that are of low importance and have a low performance level. However, these factors may not require further improvement.

High priority (Quadrant IV): This quadrant includes factors that are highly important and have low performance. These factors require immediate attention and should be prioritized for improvement.

By identifying the four-quadrant partitions, stakeholders can prioritize their efforts on the most important factors that have the greatest potential for improvement and make the most effective use of limited resources. We conducted an empirical IPA to assess the perceived importance of vertical violence in the workplace among nursing interns. The violence problem corresponding to the four different characteristic categories of IPA was analyzed to determine the improvement area.

## Results

### Data collection and participants

The nursing interns involved in this study were all from a Grade A general hospital in Zhejiang Province and completed a questionnaire survey in February 2023. In total, 130 questionnaires were sent out and 120 were effectively received, with a recovery rate of 92.3%. Men and women accounted for 7 and 93% of the respondents, respectively. Over 63.3% of nursing interns were under the age of 21 years, of which 82.5% had practiced for more than 30 weeks and were about to complete their clinical practice. Table 2 presents participants’ information. In this study, the reliability coefficient of tool’s Cronbach’s alpha was *r* = 0.949.

**Table 2:** Nursing interns’ demographic characteristics (*n* = 120).

Items	*n*	%
Gender		
Male	8	7%
Female	112	93%
Age (year-old)		
≤21	76	63%
22–23	41	34%
≥24	4	3%
Internship time (week)		
≤30	21	18%
31–39	80	67%
≥40	19	15%
Education level		
Junior college education	9	7%
Undergraduate education	111	93%

### The results of IPA

The importance and performance of vertical violence in the workplace of nursing interns are represented by the *x* and *y* axes of the IPA chart, respectively. Table 3 lists the importance and performance of the 15 violence-related items based on the questionnaire. According to the IPA chart, all the standards can be divided into the following four areas:

**Table 3:** IPA results of the workplace vertical violence of nursing interns (*n* = 120).

Items	Importance	Performance	Quadrant
*C*_1_	3.558	3.942	IV
*C*_2_	3.550	3.908	IV
*C*_3_	3.617	3.842	IV
*C*_4_	3.325	4.117	II
*C*_5_	3.492	4.058	II
*C*_6_	3.475	4.158	II
*C*_7_	3.500	4.200	I
*C*_8_	3.575	4.017	IV
*C*_9_	3.558	3.975	IV
*C*_10_	3.508	4.158	I
*C*_11_	3.517	4.058	I
*C*_12_	3.450	3.808	III
*C*_13_	3.433	4.058	II
*C*_14_	3.500	4.133	I
*C*_15_	3.425	3.975	III
*Center*	3.498	4.027	

**Figure 2:**
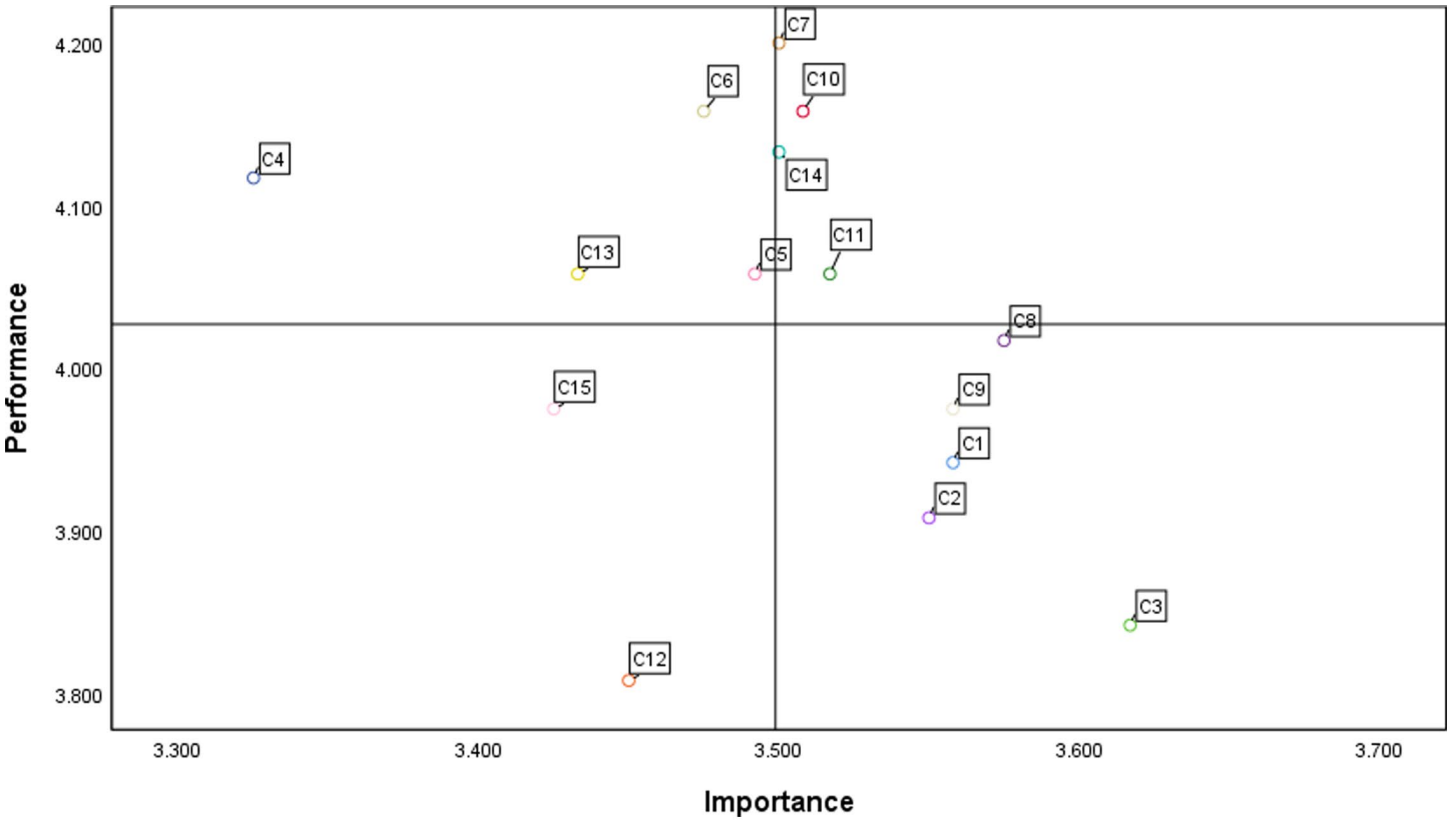
The quadrants diagram based on the IPA method.

Keep up the good work (quadrant I) includes “(*C*_7_),” “(*C*_10_),” “(*C*_11_),” “(*C*_14_).” Possible overkill (Quadrant II) includes “(*C*_4_),” “(*C*_5_),” “(*C*_6_),” “(*C*_13_).” Low priority (Quadrant III) includes “(*C*_12_),” “(*C*_15_).” High priority (Quadrant IV) includes “(*C*_3_),” “(*C*_8_),” “(*C*_9_),” “(*C*_1_),” and “(*C*_2_),” which are the crucial variables determining the workplace vertical violence of nursing interns. Simultaneously, they are priority improvement variables. Detailed results are presented in Figure 2.

## Discussion

### The crucial variables

This study highlights the prevalence of vertical violence among nursing interns in the workplace (19, 23). The prevalence of workplace vertical violence in the nursing profession is a concern, as evidenced in our study (7). The most frequently encountered workplace vertical violence to nursing interns in our study were concluded as follows: “(*C*_3_),” “(*C*_8_),” “(*C*_9_),” “(*C*_1_),” and “(*C*_2_).” The results of type importance in the above 5 showed that it was a key variable in determining vertical violence in the workplaces of nursing interns. At the same time, they are priority improvement variables.

Given the lack of research on vertical violence among nursing interns in the workplace, this issue is rarely addressed in clinical practice. Nursing interns often have a limited understanding of vertical violence and some students are inexperienced, which may not fully reflect the types of violence that occur in the workplace. Furthermore, the types of workplace vertical violence included in this study may be inadequate, leading to an overestimation of the prevalence of violence experienced by nursing interns.

However, our results are consistent with existing literature on violence. In 2019, Tian et al. (7) surveyed 249 nursing interns regarding vertical violence at their workplace. Among them, 55.4% of nursing interns were ordered to do something beyond their abilities and without guidance. 41.8% of nurses were unfairly criticized; 37.8% of nursing interns were repeatedly stressed, propagated, or their work errors were exaggerated. Approximately one-third of nursing interns were devalued at work or had information withheld. Furthermore, Fernandez-Gutierrez and Mosteiro-Diaz (31) found that students often suffered from the aforementioned behaviors, which made them feel worthless. Karatas et al. (32) surveyed 202 nursing interns, 9% of whom were assigned impractical workload. These types of bullying are also documented in many quantitative studies (2, 33). Smith et al. (18) reported different expression forms, constituting six types of bullying among respondents: being ignored or isolated, negative nonverbal behavior, interacting negatively with students and spreading rumors, refusing learning opportunities, feeling embarrassment in front of other professionals or patients, and being intimidated or threatened.

### The priority improvement variables

The importance results showed that “(*C*_3_),” “(*C*_8_),” “(*C*_9_),” “(*C*_1_),” and “(*C*_2_)” were crucial variables in determining workplace vertical violence among nursing interns and are priority improvement variables. Based on the results of the empirical questionnaire survey and the IPA method, we sorted the high-priority regional projects and discussed the corresponding intervention measures by topic.

*“I was ordered to do something beyond my ability and lacked guidance (C_3_).”*

Nursing interns are often ordered to do more than they can handle without guidance. This problem may be due to a shortage of nursing staff (34), high workload, high responsibility (35), and stress (36), such that nurses may force students to complete the heavy work they should do. Adequate staffing can prevent students from becoming overburdened, and enable them to receive guidance when needed. Therefore, we should actively protect the number of nursing staff, improve the working environment, and solve the problems of nursing staff allocation, supply and demand, and turnover caused by a shortage of nurses and poor working conditions, which are important causes of vertical violence in the workplaces of nursing interns (37). Senior nurses, who are likely to be sources of vertical violence among nursing interns, should be educated on the subject. Interventions to support senior nurses in managing the work stress they may experience should be designed (38). At the same time, feedback meetings or simulated training courses can be set up so that students can reflect on their own behavior and recognize problems so that they can actively learn to avoid the occurrence of violence.

*“Errors in work have been repeatedly emphasized, spread, or exaggerated (C_8_).”*

In clinical work, mistakes made by interns will be repeatedly emphasized by teachers, and even exaggeration phenomena will occur frequently, although teachers hope to remind students that similar mistakes should not occur again. However, the nursing interns were distressed. To improve situations where mistakes made by interns are repeatedly emphasized and exaggerated by teachers, it is important for managers to provide a supportive and non-punitive learning environment to nursing interns during their clinical practice. Managers are advised to foster an atmosphere that emphasizes learning from mistakes rather than blaming or punishing interns. Thomson et al. (39) argues that positive attention is attractive and powerful to participants and is important for creating a blame-free environment.

In a large percentage of cases, students did not know where or how to report these types of cases. When they became aware of violence, only one in five students voluntarily reported the incident (17). Nursing interns do not often report this situation because they do not feel empowered to do so and find mentors inaccessible (33). Therefore, it is necessary to provide nursing interns with opportunities to report and reflect on their practice (40). Studies have shown that debriefing after critical incidents or near-misses can help nursing interns learn from their experiences and identify areas of improvement (41). The creation of new or revised policies is one of the most common workplace responses to bullying. However, policy alone does not bring any change. Only by disseminating professional values can complaints be legitimized and complaint procedures clarified (42).

*“I was unjustly criticized (C_9_).”*

Nursing interns often receive unfair criticism during practice. Several strategies can be implemented to improve situations where nursing interns receive unfair criticism during practice. First, clinical educators and preceptors should receive training to provide constructive and supportive feedback rather than critical or negative comments (43). Second, promoting a culture of learning and continuous improvement in the clinical setting can encourage nursing interns to reflect on their experiences and use these reflections as a basis for self-directed learning and improvement (44). This can be achieved through reflective-writing exercises, self-assessment tools, and individual coaching and mentoring.

Finally, it is important for clinical educators and preceptors to recognize the potential impact of unfair criticism on nursing interns and provide support and resources to address any negative effects. This can include access to counseling and mental health services, as well as opportunities for debriefing and peer support. Furthermore, we found that students reported levels of anxiety and depression that were higher than that in the general population (45); 47.2 and 54% did not seek support for above-threshold anxiety and depression, respectively (46). Therefore, mental health service information should be made readily available to nursing interns. By implementing these strategies, clinical educators and preceptors can create positive learning environments promoting the development of clinical skills and knowledge.

It is essential to provide constructive feedback to interns in a respectful and supportive manner. This approach can help interns understand their mistakes and develop plans to improve their performance without feeling demoralized or demotivated. It is also crucial for supervisors to acknowledge the interns’ strengths and positive contributions, which can boost their confidence and self-esteem (47, 48).

*“I was withheld or blocked information purposefully (C_1_).”*

To improve situations where nursing interns feel that information is deliberately withheld or blocked, it is imperative for clinical educators and preceptors to establish clear communication and provide adequate support for their students. Research has demonstrated that good communication and support can improve mental resilience. This promotes a positive learning environment that facilitates the acquisition of clinical skills and knowledge (42). Additionally, clinical educators and preceptors must recognize the importance of student empowerment and engagement in clinical setting (49). Nursing interns should be encouraged to play an active role in their learning and be given opportunities to contribute to patient care and decision-making processes.

*“I was belittled at work (C_2_).”*

To improve interns’ feelings of undervaluation at work, supervisors must provide regular feedback and recognize their work. Studies have shown that positive feedback and recognition can increase job satisfaction and motivation among nursing interns in clinical practice. One study found that nursing interns assigned more responsibility and autonomy in clinical practice reported greater job satisfaction and motivation. The authors suggested that supervisors should consider giving interns more opportunities to take on challenging tasks and actively involve them in patient care decisions (50).

Nursing interns experience other varied behaviors at the workplace, as identified in this study. Therefore, nursing educators and administrators should focus on supporting and protecting vulnerable nursing interns from violence. This can be achieved by implementing appropriate policies and regulations to prevent and manage workplace violence (51). According to the types of workplace violence discovered in this study, targeted training plans for violence prevention should be designed for nursing teachers and students, such as anti-bullying training situational simulation (16, 52) and resilience courses (53, 54). A multifaceted and sustained intervention approach may be effective in such cases. By raising awareness on bullying among supervisors and registered nurses, employees can better evaluate their own behaviors (33) and minimize the occurrence of vertical violence in the workplace, ultimately improving the working environment for nursing interns.

The nursing environment is very heavy, and the patient’s condition is different every day. Nursing ability and experience need time to accumulate. However, for nursing interns, they may feel physically tired when they first face nursing work, which may make these experiences psychologically sensitive. Therefore, in nursing work, we should pay special attention to whether interns have undertaken work beyond their ability; on the psychological level, we should pay attention to whether interns feel that the work is unfair and whether they have a sense of accomplishment.

### Research limitations

This study has some limitations. First, the questionnaire on vertical violence in the workplace for nursing interns in this study was compiled by researchers based on previous literature, without using a standard scale. Second, our study adopted a cluster sampling method and was limited to one hospital, so there may have been selection bias in the data analysis. This may have affected the representativeness of the survey results. Therefore, to determine greater typicalness, a scale with good reliability and validity should be adopted to conduct a cross-sectional random sampling survey, and the data sample should cover multiple hospitals in various provinces across the country. Third, the cross-sectional nature of our study means we can only draw conclusions about the associations between variables at a single point in time. We cannot infer causality or changes over time. Longitudinal studies would provide further insights into the temporal relationships between the variables we investigated. Fourth, our use of self-reported questionnaires might introduce response bias. Participants may have over-or under-reported their experiences with workplace vertical violence due to social desirability bias or recall bias. Future studies might benefit from using multiple data sources or methods to validate the findings. Despite these limitations, we believe that our study contributes valuable insights into the understanding of workplace vertical violence among nursing interns. Future research is needed to confirm and extend our findings. Fifth, the premise of using IPA method is to ask about the importance and satisfaction of each item. In this study, each interviewee was investigated in the form of the questionnaire.

## Conclusion

To create a more conducive internship environment, supervisors must strategically deploy resources to minimize false reinforcement and unwarranted criticism. Emphasizing the exchange of information, highlighting the clinical roles of nursing interns, and providing judicious guidance when tasks exceed their professional capacities are key. Additionally, efforts should be made to mitigate workplace vertical violence and to foster an improved clinical environment for nursing interns. Moving forward, further research is needed to understand the mechanisms and long-term impacts of such violence. The insights from this study serve as a valuable guide for the development of effective interventions aimed at enhancing the workplace experience of nursing interns and improving the quality of patient care.

## Data availability statement

The original contributions presented in the study are included in the article/Supplementary material, further inquiries can be directed to the corresponding authors.

## Ethics statement

All procedures were performed in accordance with the guidelines of the Ethics Committee of the Zhejiang Taizhou Central Hospital (Approval Number: 2023 L-02-01). Written informed consent from the participants was not required to participate in this study in accordance with the national legislation and the institutional requirements.

## Author contributions

WX, MY, and Y-CC wrote the draft of the article. HH collected the data. Y-CC calculated the IPA results of this study. LF and SL were responsible for the discussion and analysis of the results of this study. FT, FC, and Y-CC directed the whole study. All authors agreed with the above expression of author’s contribution.

## Funding

This work was supported by the Zhejiang Medical and Health Science and Technology Program (No. 2023KY1337), the Education Planning Project of Taizhou City, Zhejiang Province (Nos. GG22016 and GG22019), and Nursing Discipline Development Special Fund Project of Taizhou University, Zhejiang Province (No. 202201).

## Conflict of interest

The authors declare that the research was conducted in the absence of any commercial or financial relationships that could be construed as a potential conflict of interest.

## Publisher’s note

All claims expressed in this article are solely those of the authors and do not necessarily represent those of their affiliated organizations, or those of the publisher, the editors and the reviewers. Any product that may be evaluated in this article, or claim that may be made by its manufacturer, is not guaranteed or endorsed by the publisher.
