# Supplementary material for: Evaluation and improvement of workplace vertical violence of nursing interns based on the Importance-Performance Analysis method
Source: Front Med (Lausanne). 2023 Sep 28;10:1210872. doi: 10.3389/fmed.2023.1210872 (PMC10569029; doi:10.3389/fmed.2023.1210872)
Supplement: Supplementary file 1 [file Data_Sheet_1.docx]

**Table 1 Workplace Vertical Violence Measurement Scale for nursing interns**

| Question item | Very unimportant  (1 points) | Unimportance  (2 points) | Generally important  (3 points) | Importance  (4 points) | very important  (5 points) |
| --- | --- | --- | --- | --- | --- |
| 1. I was withheld or blocked information purposefully (*C*_1_) |  |  |  |  |  |
| 1. I was belittled at work (*C*_2_) |  |  |  |  |  |
| 1. I was ordered to do something beyond my ability and lacked of guidance (*C*_3_) |  |  |  |  |  |
| 1. Others spread gossip or rumors about me (*C*_4_) |  |  |  |  |  |
| 1. I was frozen out, ignored, or excluded (*C*_5_) |  |  |  |  |  |
| 1. I was threatened or intimidated (*C*_6_) |  |  |  |  |  |
| 1. I was humiliated publicly (*C*_7_) |  |  |  |  |  |
| 1. Errors in work have been repeatedly emphasized, spread, or exaggerated (*C*_8_) |  |  |  |  |  |
| 1. I was unjustly criticized (*C*_9_) |  |  |  |  |  |
| 1. I was physically abused (such as pushing body behavior) (*C*_10_) |  |  |  |  |  |
| 1. I was deprived of proper rights (*C*_11_) |  |  |  |  |  |
| 1. I was a laborer who was forced to do trivial and unimportant work (*C*_12_) |  |  |  |  |  |
| 1. I was treated with hostility (*C*_13_) |  |  |  |  |  |
| 1. I became a scapegoat (*C*_14_) |  |  |  |  |  |
| 1. Turn to others for help, but they refused to help. (*C*_15_) |  |  |  |  |  |

Part two: Violence performance

| Question item | Completely agree  (1 points) | Agree  (2 points) | General agreement  (3 points) | Disagree  (4 points) | Completely disagree (5points) |
| --- | --- | --- | --- | --- | --- |
| 1. I was withheld or blocked information purposefully (*C*_1_) |  |  |  |  |  |
| 1. I was belittled at work (*C*_2_) |  |  |  |  |  |
| 1. I was ordered to do something beyond my ability and lacked of guidance (*C*_3_) |  |  |  |  |  |
| 1. Others spread gossip or rumors about me (*C*_4_) |  |  |  |  |  |
| 1. I was frozen out, ignored, or excluded (*C*_5_) |  |  |  |  |  |
| 1. I was threatened or intimidated (*C*_6_) |  |  |  |  |  |
| 1. I was humiliated publicly (*C*_7_) |  |  |  |  |  |
| 1. Errors in work have been repeatedly emphasized, spread, or exaggerated (*C*_8_) |  |  |  |  |  |
| 1. I was unjustly criticized (*C*_9_) |  |  |  |  |  |
| 1. I was physically abused (such as pushing body behavior) (*C*_10_) |  |  |  |  |  |
| 1. I was deprived of proper rights (*C*_11_) |  |  |  |  |  |
| 1. I was a laborer who was forced to do trivial and unimportant work (*C*_12_) |  |  |  |  |  |
| 1. I was treated with hostility (*C*_13_) |  |  |  |  |  |
| 1. I became a scapegoat (*C*_14_) |  |  |  |  |  |
| 1. Turn to others for help, but they refused to help. (*C*_15_) |  |  |  |  |  |
